# The role of travel for people with an eating disorder, an optimal leisure experience

**DOI:** 10.1192/j.eurpsy.2021.1851

**Published:** 2021-08-13

**Authors:** L. Mostefa-Kara

**Affiliations:** Cognitive Sciences, Research center of the Paul Bocuse Institute, Lyon, France

**Keywords:** eating disorder, travel, digital tools, optimal experience

## Abstract

**Introduction:**

Project-based leisure would be conducive to enabling people to project themselves into the future and to move away from the eating disorder, which involves a constant preoccupation with food and one’s body. According to this, travel could be a leisure opportunity that invites to challenge oneself by going out of one’s comfort zone. Coping is a real dilemma for people living with eating disorders and the motivations of a tourism experience could refer to motivation to leave one’s current environment. Consequently, travel would be helpful in reducing the individual’s focus on the illness in a different environment.

**Objectives:**

The aim of this study is to investigate the use of travel to help people living with eating disorders to live in the present moment and to “let go”. Then, to understand what are the components of travel that are essential for an optimal leisure experience.

**Methods:**

This multiple case study uses mixed data from a sample of five participants with an ED and living in France. They were invited to live a tourism experience in Québec for one week. This data collection was before, during and after the trip, using the Experience Sampling Method with a mobile app pocket and guided interviews to assess sensations and emotions in the ecological context of patients.

**Results:**

The trip allowed a letting go and developed a greater ability to live in the present moment. It was a significantly positive moment in the lives of the participants.

**Conclusions:**

Travel associate with digital diary are an innovative approach for ED.

**Disclosure:**

No significant relationships.

